# An intracranial extramedullary hematopoiesis in a 34-year-old man with beta thalassemia: a case report

**DOI:** 10.1186/1752-1947-5-580

**Published:** 2011-12-19

**Authors:** Homayoun Tabesh, Ahmad Shekarchizadeh, Parvin Mahzouni, Mojgan Mokhtari, Saeid Abrishamkar, Salman Abbasi Fard

**Affiliations:** 1 Department of Neurosurgery, Al-Zahra Hospital, Isfahan University Of Medical Sciences, Isfahan, Iran; 2 Department of Neurosurgery, Kashani Hospital, Isfahan University Of Medical Sciences, Isfahan, Iran; 3 Department of Pathology, Al-Zahra Hospital, Isfahan University Of Medical Sciences, Isfahan, Iran; 4 Department of Pathology, Kashani Hospital, Isfahan University Of Medical Sciences, Isfahan, Iran

## Abstract

**Introduction:**

Extramedullary hematopoiesis occurs in approximately 15% of cases of thalassemia. Intracranial deposits of extramedullary hematopoiesis are an extremely rare compensatory process in intermediate and severe thalassemia.

**Case presentation:**

We present an unusual case of an intracranial extramedullary hematopoiesis with a choroid plexus origin in a 34-year-old Caucasian man with beta thalassemia intermedia, who presented with the complaints of chronic headache and rapid progressive visual loss.

**Conclusion:**

An intracranial extramedullary hematopoiesis, although extremely rare, should be considered as a potential ancillary diagnosis in any thalassemic patient and therefore appropriate studies should be performed to investigate the probable intracranial ectopic marrow before any surgical intervention.

## Introduction

Extramedullary hematopoiesis (EMH) occurs commonly in patients with severe thalassemia who receive inadequate treatment. In this condition, beside the usual regions of hematopoiesis, blood cells can be formed in unusual sites like the liver, spleen and lymph nodes to meet the demands of hematopoiesis [1,2]. There are a few reports where EMH has involved some rare places such as the perirenal and paravertebral region, paranasal sinuses, clivus, meninges, spinal and epidural spaces, prostate, adrenals, pleura, breast, thymus, kidney, sweat gland, broad ligament and retroperitoneal space [2-11]. This unusual phenomenon, especially when it involves the central nervous system (CNS) can act as a space-occupying lesion and lead to neurological deficits [12-15]. In this report, we describe a rare case of intracranial EMH with choroid plexus origin in a patient with beta thalassemia intermedia, which involved the left occipital horn and resulted in rapid progressive visual loss.

## Case presentation

A 34-year-old Caucasian man, with known beta thalassemia intermedia, presented with the complaints of chronic headache during the last four months, with recent onset nausea, vomiting, disequilibrium and rapid progressive visual loss.

He had pallor of the skin with a cachectic appearance and a prominent skull deformity with hepatomegaly. Neurological examination showed bilateral severe optic disc atrophy and his visual acuity revealed no light perception (NLP) in both eyes. His extraocular movements and examination of his other cranial nerves were normal. His speech and sensorium were intact but there was gait disturbance due to disequilibrium. His Glasgow Coma Score was 15 and there were no paresis in his extremities. His deep tendon reflexes and cerebellar functions showed no abnormality and Babinski's sign was absent. Our patient had irregular blood transfusions, a splenectomy and cholecystectomy in his past medical history. He did not consume any kind of medicine. A family history revealed that his parents were both carriers (minor thalassemia).

On admission, a sample of his blood sent for laboratory investigations. The results showed hemoglobin 8 g/dL, white blood cell count 13.2 × 10^9^/L, platelet count 265 × 10^9^/L and mean corpuscular volume 68.9fl. A peripheral smear showed anisopoikilocytosis, tear drop cells, polychromasia, nucleated red blood cells and target cells. A hemoglobin electrophoresis showed 96.7% fetal hemoglobin and 3.3% hemoglobin alpha 2. Adult hemoglobin was absent.

**Figure 1:**
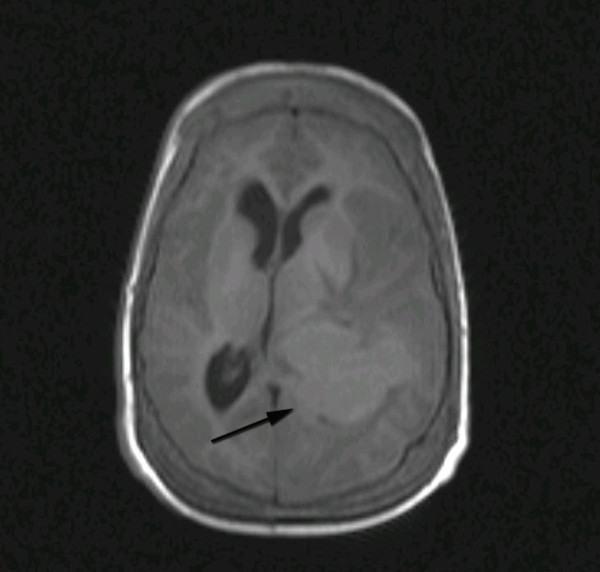
**Axial T1-weighted MRI image demonstrating a well-demarcated isointense lesion in the left trigone with significant surrounding edema and mass effect**.

**Figure 2:**
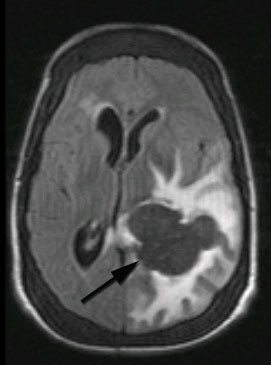
**Axial T2-weighted MRI image showing a signal void brain lesion in the left trigone area**. The surrounding vasogenic edema is well demonstrated.

**Figure 3:**
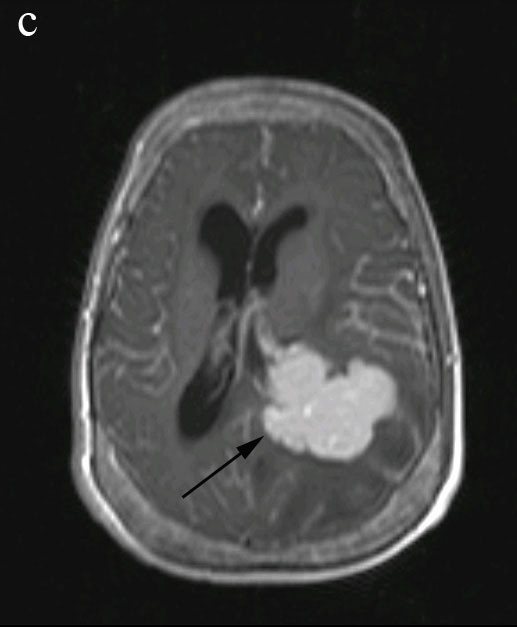
**Axial T1-weighted MRI image with IV contrast demonstrating a large homogenously enhanced lesion in the left trigone area**.

In a skull X-ray, expansion of the diploic space with a so-called hair-on-end appearance inline with EMH was seen. Computed tomography (CT) study of his brain disclosed a large hyperdense lobulated lesion in the vicinity of the left occipital horn with significant surrounding edema which enhanced after intravenous contrast injection. Magnetic resonance imaging (MRI) of his brain revealed a hyperintense lesion in T1-weighted images which was signal void in T2-weighted imaging, with significant peripheral edema (Figures 1, 2 and 3). There was a left-to-right shift of the midline and thickening of the calvarium in the sagittal plane.

After correcting the hemoglobin level before the operation, with the impression of a meningioma of a lateral ventricle or high grade glioma, a craniotomy was performed, using a left posterior parietal transcortical approach.

Intraoperatively, the lesion was found to be lobulated, very well differentiated from the peripheral white matter without adhesion to it and well capsulated with prominent vessels over the capsule of the tumor. The lesion was excised entirely up to its final attachment to the choroid plexus, which came in to view, and the occipital horn was seen.

**Figure 4:**
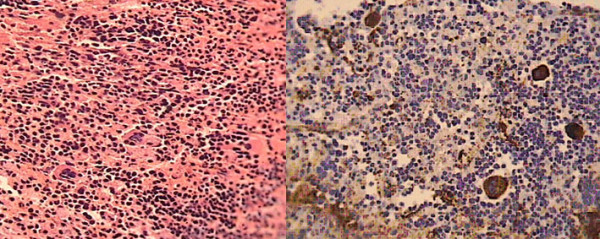
**Microscopic view of the excised brain lesion**. Demonstrates **(A) **the infiltration by foci of hematopoietic cells and megakaryocytes (hematoxylin and eosin stain, original magnification ×40) and **(B) **megakaryocytes and endothelial cells positive for factor VIII related antigen (hematoxylin and eosin stain, original magnification ×40).

A pathologic examination of the surgical specimen was compatible with hematopoietic tissue. Macroscopically, the specimen measured 2.5 cm×1.5 cm×1 cm and was soft and grayish white. Microscopically, the specimen was composed of brain tissue, hematopoietic cells and megakaryocytes (Figure 4A). On immunohistochemistry, the megakaryocytes and endothelial cells were positive for factor VIII related antigens (Figure 4B). Postoperatively, our patient was followed for four months. He showed rapid recovery from the signs and symptoms of increased intracranial pressure but his recovery was incomplete with regards to his visual loss, that is, his visual acuity was improved sufficiently to detect hand motion. During this period, our patient was referred to a hematologist and radiotherapist for continuation of the standard treatment.

## Discussion

Spinal cord compression due to EMH is a well-known condition, however intracranial involvement is extremely rare and there are few reported cases about this disorder [13,15]. EMH is described as an ectopic production of bone marrow elements, which is believed to be a compensatory mechanism subsequent to a body's hematologic demands and bone marrow stress [16]. In some conditions, this ectopic mass is as a result of bone marrow extension from the adjacent bony structures. A variety of modalities have been advocated to relieve the bone marrow compression [8,15]. Multiple blood transfusions and low-dose radiation are some of these proposed treatments, which can lead to shrinkage of the ectopic marrow and a decline in compression signs and symptoms.

In our case, our patient had symptoms of increased intracranial pressure and visual loss. The latter symptom may have been a secondary effect of the mass lesion upon adjacent structures of his visual pathways and/or increased intracranial pressure. Moreover, an intracranial EMH arising from the base of the cranium near the sphenoid and ethmoid bones may extend into the intracranial cavity and compress the visual pathways, with resultant progressive visual failure [15].

In one previous case report, Aarabi and colleagues presented a similar case of an intracranial EMH in a thalassemic patient with symptoms of decreased visual acuity. They also surgically excised the intrasellar mass, but their patient had a previously determined EMH arising from the base of his cranium near the sphenoid and ethmoid bones, extending into the intracranial cavity. Their management included partial resection of the mass [15].

Musolino and his colleagues also reported a similar case of an intracranial EMH with symptoms of decreased visual acuity and endocranial hypertension, but their patient had acquired immune deficiency syndrome with chronic bone marrow dysfunction. It was mentioned that the decreased visual acuity was due to cytomegalovirus chorioretinitis, contrary to that seen in our case which was due to a compressive effect of the intracranial EMH [13].

An interesting finding in the current case report was that we were presented with a rare case of an intracranial EMH originating from the choroid plexus, and our patient underwent successive total surgical resection and the signs and symptoms of increased intracranial pressure subsided. Although total resection of this lesion is a hemorrhagic procedure, it seems that, in operable cases, total resection may be safe and help with the early remission of the clinical signs and symptoms.

## Conclusion

This case report showed that although rare, an intracranial EMH should always be considered in a differential diagnosis of any mass lesion and increased intracranial pressure symptoms in patients with thalassemia. Therefore, appropriate investigations should be performed to diagnosis the probable intracranial ectopic marrow lesion before any surgical intervention.

## Consent

Written informed consent was obtained from the patient for publication of this case report and any accompanying images. A copy of the written consent is available for review by the Editor-in-Chief of this journal.

## Competing interests

The authors declare that they have no competing interests.

## Authors' contributions

SAF performed the chart review and manuscript preparation. HT, ASH, SA and SAF carried out the operation. PM and MM were the pathologists who examined the specimen. All authors read and approved the final manuscript.
